# *Hypsilara royi* gen. n. and sp. n. (Coleoptera, Elmidae, Larainae) from Southern Venezuela, with a revised key to Larainae of the Western Hemisphere

**DOI:** 10.3897/zookeys.116.1347

**Published:** 2011-07-07

**Authors:** Crystal A. Maier, Paul J. Spangler

**Affiliations:** 1 Division of Entomology, Biodiversity Institute & Department of Ecology and Evolutionary Biology, University of Kansas, Lawrence, KS, 66045, USA; 2 Department of Entomology, National Museum of Natural History Smithsonian Institution, Washington, D.C., 20560, USA

**Keywords:** Aquatic insects, Cerro de Neblina, Neotropical Region, riffle beetle, tepui, Guiana Shield

## Abstract

Here we describe a new genus, for a new species of riffle beetle, *Hypsilara royi* **gen.** **n.** and **sp. n.**, from the tepui Cerro de la Neblina in southern Venezuela. This new genus can be distinguished from all other laraine genera by its small size (ca. 4.5 mm) and the presence of a shallow, wide, V-shaped groove across the apical third of the pronotum. An updated key to the genera of Western Hemisphere Larainae is provided, along with information on habitat and collection methods for this taxon.

## Abstract (es)

Se describe un género nuevo,  para una especie nueva de Elmidae, *Hypsilara royi* **gen.** **n. **and** sp.** **n.**, del tepui Cerro de la Neblina en el sur de Venezuela. Este género nuevo se distingue de todos los otros géneros de Larainae por su tamaño pequeño (aprox. 4.5 mm) y por la presencia de un surco superficial en forma de “V” en el tercio apical del pronoto. Se presenta una clave actualizada de los géneros de la subfamilia Larainae en el hemisferio occidental, así como información del hábitat y métodos de colecta de este taxón.

## Introduction

The Elmidae are a cosmopolitan family of beetles common in a variety of running water habitats. They are known for their “leggy” appearance, with long legs and claws that aid in clinging to rocks and other detritus in fast-flowing water. Members of the subfamily Larainae are atypical among the riffle beetles, as the adults do not live submerged, but on water-splashed rocks and in moist detritus at the water’s edge.

Currently, there are 26 genera of laraines described, with ten occurring in the Neotropical Region. The Central American and West Indian fauna was revised in 1991 by Spangler and since then, two other genera have been described from the tepuis of southern Venezuela, *Roraima* Kodada and Jäch, from Mount Roraima, and *Neblinagena* Spangler, from Cerro de Neblina.

**Figure 1.**
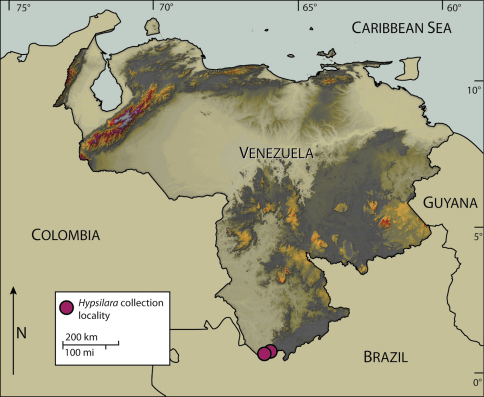
Map of Venezuela, showing collection localities for *Hypsilara* spp.

Specimens of the new genus described here were collected from Cerro de la Neblina, the “Mountain of the Mists” in southeastern Amazonas State, Venezuela (Fig. 1). Cerro de la Neblina is a 647 km2 precipitous sandstone mesa or tepui, one of numerous high, table-top mountains that occur in northern South America (Spangler 1985). These mesas are the eroded remains of a former large plateau, the Guiana Highland Shield and rise sharply from the tropical rain forests, with their tops often obscured by a dense cloud cover.

## Methods

Specimens of this new taxon were collected during a biological survey of Cerro de la Neblina in 1985. They were photographed using a Canon EOS 70D with a Visionary Digital imaging system and photos were stacked using CombineZM image editing software.

For scanning electron micrographs, the specimen was dehydrated in 100% ethanol and cleaned with an insect pin and fine brush. The specimen was then affixed to an SEM stub using carbon tape and coated with gold. Micrographs were taken using a Leo 1550 Scanning Electron Microscope at the Microscopy and Analytical Imaging Laboratory at the University of Kansas.

The genitalia were extracted from relaxed specimens through the caudal opening in the abdomen. The genitalia were then cleared in heated potassium hydroxide for thirty minutes, rinsed with water, and temporarily mounted in glycerin for observation and illustration. The cleared genitalia were then placed in a glass genitalia vial below the specimen for storage.

Hind wings were removed and dry mounted on slides using methods outlined in Kukalová-Peck and Lawrence (1993). Veins were homologized using terminology from Kukalová-Peck and Lawrence (1993) and Kodada and Jäch (2005). Illustrations were made in pen and ink using a camera lucida attached to a Olympus SZX7 microscope. Images were edited in Adobe Illustrator and Adobe Photoshop. Specimens were deposited in the following collections:

MIZA	Museo del Instituto de Zoología Agrícola Maracay, Venezuela

MALUZ	La Universidad del Zulia, Maracaibo, Venezuela

NMPC	National Museum, Prague, Czech Republic

NMW	Naturhistorisches Museum, Vienna, Austria

SEMC	Snow Entomology Collection, University of Kansas, Lawrence, Kansas, USA

USNM	Smithsonian Institution, Washington, DC, USA

## Taxonomy

### Hypsilara gen. n.

urn:lsid:zoobank.org:act:0F2AFFF1-1D67-4FDD-BE59-1151F6926584

http://species-id.net/wiki/Hypsilara

**Figures 2–4.**
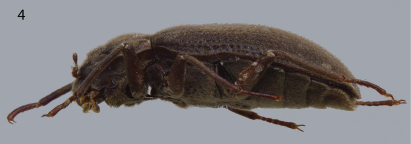
*Hypsilara royi* sp. n. **2** Habitus, dorsal view; Scale bar = 2 mm **3** Habitus, ventral view **4** Habitus, lateral view.

Figs 2 [Fig F3] [Fig F4] 17 

#### Type species:

*Hypsilara royi* sp. n.

#### Diagnosis.

This genus can be distinguished from all other laraine genera by its small size (ca. 4.5 mm), and the presence of a shallow, wide, V-shaped groove across apical third of the pronotum (Fig. 10).

#### Description.

Body elongate, form gradually widening to posterior two-thirds of elytra then converging to elytral apex, moderately convex (Fig. 2). Integument clothed with dense, recumbent pubescence.

Head capable of being partly retracted into prothorax but not beyond the basal portion of the submentum (Fig. 4). Maxillary palpus four segmented. Labial palpus three segmented. Antenna eleven segmented, with apical six segments forming a club (Fig. 8). Clypeus transversely subrectangular; frontoclypeal suture deeply impressed between bases of antennae; anterolateral angles broadly rounded. Labrum transversely subrectangular; anterior margin without distinct emargination medially; anterolateral angles broadly rounded.

Pronotum widest at base, weakly sinuate laterally, becoming evenly arcuate over head; base trisinuate, broadly sinuate on each side and much more narrowly so immediately anterior to scutellum; anterolateral angles explanate and broadly depressed; posterolateral angles slightly explanate, declivous, not depressed; middle of base with two short, broad, prescutellar cariniform ridges, each ridge with distinct lateral depression (Fig. 10). Pronotum with V-shaped discal groove; lateral branches of groove shallowly depressed, almost confluent with sublateral arcuate-sinuate groove but interrupted by short carina laterally; stem of Y-shaped groove shallow; sublateral carina short, evident at base then merging with lateral margin. Scutellum flat, wider than long, subtriangular. Elytron with ten longitudinal rows of deep punctures; without accessory row of punctures; without complete longitduinal carinae; apex rounded, not prolonged (Fig. 11). Prosternum very long anteriorto procoxae, about as long as prosternal process; moderately reflexed along anterior margin (Fig. 3). Prosternal process broadly triangular between procoxae; apex narrowed and rounded. Mesoventrite with deep, broadly V-shaped depression on midline for the reception of apex of prosternal process. Metaventrite with disc shallowly, broadly depressed on posterior two-thirds; with longitudinal groove deepest and broadest on posterior third of midline (Fig. 14). Legs with visible portion of procoxae transverse and trochantin visible. Claws prominent and without teeth (Figs 15, 16).

**Figure 5–7. F3:**
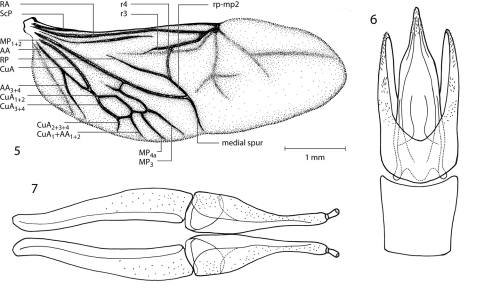
*Hypsilara royi* sp. n. **5** Hind wing **6** Male genitalia **7** Female genitalia.

Hind wing lightly pigmented. Radial bar strong; radio-medial loop and radial cross vein r4 distinct; radial cell incomplete; medial fleck absent; media posterior MP1+2 strong, distinct; medial spur long, nearly reaching wing margin; first and second cubito-anal cells present; medial field with five free veins reaching margin (medial spur not included); anal field with single vein; apical field with two lightly pigmented bands (Fig. 5).

Abdomen with five ventrites. First ventrite with paired, broad carinae posterior to metacoxae extending almost to hind margin of ventrite (Fig. 17).

**Figures 8–13. F4:**
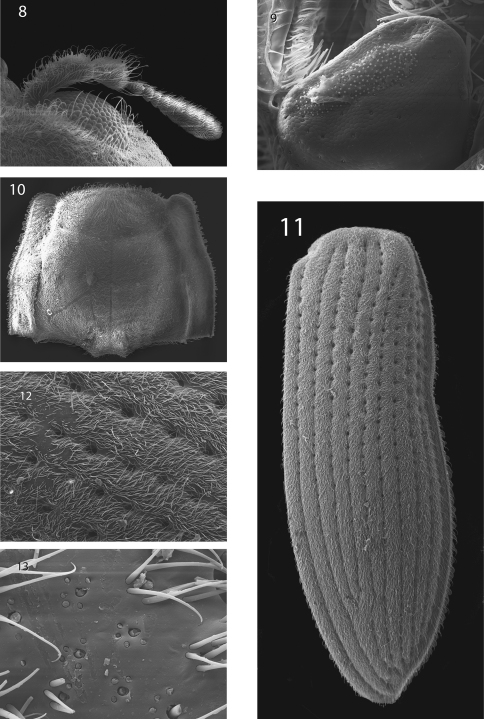
*Hypsilara royi* sp. n. **8** Antenna **9** Maxillary palp **10** Pronotum, dorsal view **11** Elytron **12** Elytron, detail **13** Elytron, setae removed to show configuration.

**Figures 14–17.**
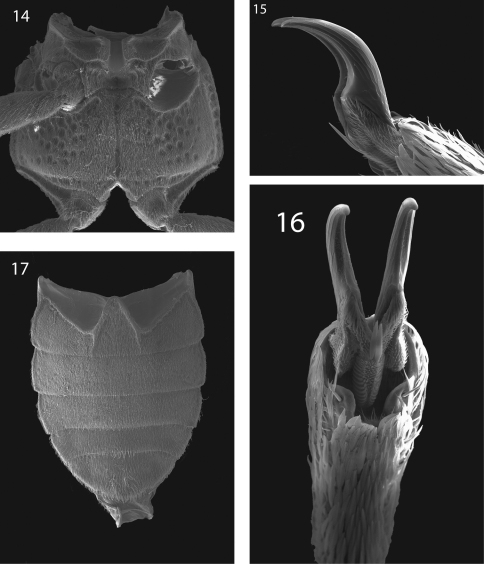
*Hypsilara royi* sp. n. **14** Metaventrite **15** Tarsal claw, lateral view **16** Tarsal claw, ventral view **17** Abdomen, ventral view.

#### Etymology.

*Hypsilara* from the Greek “hypsos” meaning “aloft”, from its elevated habitat on Cerro de Neblina, and “lara”, from the subfamily name, Larainae. The gender is feminine.

#### Remarks.

*Hypsilara royi* sp. n. was also collected from a flight intercept trap operated in the lowland rain forest (140 m elev.) near the base camp (00°50'N, 66°10'W ) (Fig. 1). PJS had designated this single male specimen as a new species; based on genitalia differences, though specimens bearing genitalia similar to the base camp specimen were collected at the type locality of *Hypsilara royi* sp. n. Therefore, we decided not to designate it as a new species at this time.

#### Comparative notes.

This new genus keys to couplet 4 in Brown’s (1981) key to the world genera of the elmid subfamily Larainae. Since Brown’s key was published, four new genera of Larainae have been described from the Western Hemisphere: *Neblinagena* Spangler, *Pharceonus* Spangler and Santiago, *Roraima* Kodada and Jäch, and *Hypsilara* gen. n. Therefore we are presenting the following revised key to the Larainae of the Western Hemisphere which incorporates all genera described to date.

#### Key to the Genera of Adult Larainae of the Western Hemisphere

**Table:** 

1	Body size smaller, length 2.5 to 4.5 mm	2
1'	Body size larger, length 5.1 to 10.1 mm	6
2(1)	Elytron with one accessory basal stria. Chile and Argentina	*Hydora* Broun, 1882
2'	Elytron without an accessory basal stria	3
3(2')	Pronotum with a deep transverse groove across apical third (see Spangler and Santiago 1992: Fig. 5)	5
3'	Pronotum without a transverse groove, or with a shallow, wide, V-shaped groove across apical third (Figs 3, 10; see Spangler and Santiago 1992: Fig. 3)	4
4(3')	Pronotum with a shallow, wide, V-shaped groove across apical third (Fig. 10). Southern Venezuela	*Hypsilara* gen. n.
4'	Pronotum without a transverse groove (see Spangler and Santiago 1992: Fig. 3). Texas, USA, south to Peru, Greater Antilles	*Phanocerus* Sharp, 1882
5(3)	Pronotum with a median groove and without small prescutellar foveae; anterolateral angles of pronotum rounded (see Spangler and Santiago 1992: Fig. 4). Costa Rica, south to Venezuela	*Pharceonus* Spangler and Santiago, 1992
5'	Pronotum without a median groove and with two small prescutellar foveae; anterolateral angles of pronotum declivous (see Spangler and Santiago 1992: Fig. 5). Mexico, south to Peru and West Indies	*Hexanchorus* Sharp, 1882
6(1')	Elytron with one accessory basal stria. British Columbia, Canada and Pacific Northwestern United States	*Lara* LeConte, 1852
6'	Elytron without an accessory basal stria	7
7(6')	Elytron with distinct longitudinal carinae (see Kodada and Jäch 1999: Fig. 28). Southern Venezuela	*Roraima* Kodada & Jäch, 1999
7'	Elytron without distinct longitudinal carinae	8
8(7')	Pronotum with a distinct transverse groove across apical third	9
8'	Pronotum without a transverse groove across apical third (see Spangler and Santiago 1992 Fig. 8). Costa Rica, south to Peru	*Disersus* Sharp, 1882
9(8)	Pronotum with a lateral longitudinal carina or arcuate-sinuate groove on basal third	10
9'	Pronotum without a carina or arcuate-sinuate groove on basal third	11
10(9)	Pronotum with two prescutellar mammiform tubercles at base and one similar tubercle near each posterolateral angle, thus appearing bidentate (see Spangler and Santiago 1992: Fig. 6). Panama, south to Ecuador	*Pseudodisersus* Brown, 1981
10'	Pronotum with two short, converging, prescutellar carinae, each with a deep pit laterally (see Spangler 1989: Fig. 9). Venezuela	*Neblinagena* Spangler, 1985
11(9')	Body narrow. Prosternal process sagittate, tapering gradually to apex (see Spangler and Santiago 1992: Fig. 215). Hispañola	*Hispaniolara* Brown, 1981
11'	Body broad. Prosternal process ligulate at apex (see Spangler and Santiago 1987: Fig. 128). Argentina and Brazil	*Potamophilops* Grouvelle, 1896

### Hypsilara royi sp. n.

urn:lsid:zoobank.org:act:F34363E2-E809-4193-8EC6-35416E0D66FF

http://species-id.net/wiki/Hypsilara_royi

Figs 2 [Fig F3] [Fig F4] 17 

#### Type material.

Holotype male: “VENEZUELA: T. F. AMAZ./ Cerro de la Neblina/ Camp XI 1450 m/ 00°52'N, 65°58'W ” “at stream/ 26–27 February 5 l985/ P. J. and P. M. Spangler, R. A. Faitoute/ collector”. Holotype deposited in MIZA. Paratypes (31): Same locality data as holotype (4 males 11 females); same data as holotype, except Camp X, 00°54'N, 60°2'W , 1690m, 12–13 February l985, W. Steiner (8 males 8 females). Paratypes will be deposited in: 7 in MIZA, 1 in MALUZ, 1 in NMPC, 1 in NMW, 5 in SEMC, and 16 in USNM.

Other Material Examined: “VENEZ., T.F.A./C.d.l. Neblina/Base camp/26–31 Jan. 1985/Flite [sic] intercept Pan/Trap” (1 male). “VENEZUELA: T. F. AMAZ./ Cerro de la Neblina/ Camp X, 1690m/ 00°54'N, 60°2'W / 12 February l985” “Small sunlit stream;/leaf packs in falls/between boulders/ W. E. Steiner,/collector” (3 females).

#### Diagnosis.

Monotypic genus – see generic diagnosis.

#### Description.

Holotype Male. Body elongate, subparallel, dorsum moderately convex. Length, 4.4 mm; greatest width, 2.0 mm. Body dark brown dorsally; venter light reddish brown, except elytral epipleura, metepisternum, lateral margins of abdominal ventrites 3–5 dark brown. Antenna, maxillary palpus, labial palpus, labium, maxilla, coxa, trochanter, basal four-fifths of profemora and mesofemora lighter reddish-brown. Dorsal and ventral surface densely covered with recumbent setae (Fig. 2).

Head moderately coarsely, densely punctate; punctures separated by their diameter; cuticle microreticulate. Eye hemispherical, bordered with row of long, curved setae (Fig. 8). Basal two segments of antenna with long, dense pubescence (Fig. 8). Clypeus anteriorly with shallow arcuate emargination. Labrum subrectangular; surface with fine, dense punctation and densely fringed with long, fine, golden, hairlike setae; anterolateral angles rounded but not expanded laterally; lateral margins not expanded, with a long, dense tuft of golden hairlike setae curled over margin. Labium with long, dense setae. Last segment of maxillary palpus broad and bearing sensillae on flattened apex (Fig. 9).

Pronotum 1.4 mm long, 1.5 mm wide; with deep sinuate sublateral groove, which extends from apical third of pronotum to base; lateral margins slightly sinuate; anterolateral angles obtuse, distinctly explanate and broadly depressed behind each angle; apex slightly sinuate and with broad lobe medially; posterolateral angles obtuse, slightly explanate, declivous, not depressed adjacent to each angle (Fig. 10); discal area with fine, dense punctures, punctures separated by a distance equal to or less than their diameter; cuticle microreticulate.

Prosternum very long in front of procoxae; bearing a tuft of sparse, long, dark brown setae and dense golden setae apicomedially. Prosternal process (Fig. 3) triangular, broad at base and tapering to apex; lateral margins reflexed; middle moderately longitudinally cariniform; apex narrow, rounded. Mesoventrite with a deep, broad, V-shaped depression for reception of apex of prosternal process. Metaventrite with disc depressed on posterior three-fourths, coarsely punctate behind mesocoxae, punctures becoming more sparse laterally, with large, rounded depressions scattered on disc (Fig. 14); with a deep, narrow, shining, longitudinal groove on midline of disc, groove deepest and broadest on posterior third of disc; with short, dense, golden pubescence and a patch of longer darker brown setae on each side of median groove on apical third; cuticular surface of metaventrite finely microreticulate. Procoxae and metacoxae moderately widely separated; mesocoxae slightly more widely separated. Legs long and slender. Protibiae, mesotibiae, and metatibiae (Fig. 3) with dense spatulate pubescence distally. Tarsal claws long and stout (Figs 15, 16).

Elytron with ten rows of coarse, very deep punctures (Fig. 11); punctures separated by a distance two times the diameter of the puncture (Fig. 12); intervals with fine, dense pubescence; each larger seta surrounded by four to seven smaller setae (Fig. 13) humeral area moderately swollen; elytra margined laterally; widening to about posterior two-thirds before converging to rounded apex.

Abdomen with five ventrites (Fig. 17). First ventrite with intercoxal process broadly, shallowly depressed and distinctly carinate adjacent to metacoxae; carinae extending longitudinally behind metacoxae for almost entire length of ventrite; cuticle densely covered with setae. Last visible ventrite broadly rounded.

Aedeagus with parameres straight and wide (Fig. 6). Median lobe of aedeagus slightly constricted distally.

#### Female.

Similar to male, except lacks the patch of longer setae apicomedially on prosternum and the patch on each side of median groove on apical third on metasternum. Genitalia as illustrated (Fig. 7).

#### Intraspecific variation.

As noted in the “Remarks” section, the shape of the parameres of the aedeagus vary from straight and wide to narrow and curved. This species exhibits only minor variations in length, which ranges from 4.2 to 4.5 mm, and varies from a medium brown to light brown in color.

#### Etymology.

The specific epithet, “royi” is a patronym named for Roy McDiarmid, herpetologist and biological coordinator for the survey of the flora and fauna of Cerro de la Neblina.

#### Habitat.

The type specimen was collected from a small, shallow brook about one to two meters wide and with occasional pools about one meter deep, with a substrate of sand, boulders, and bedrock. This small tributary originates on Cerro de la Neblina and feeds the Rio Baria, which drains most of the massif. The highwater marks and polished boulders along the stream bed indicate that in times of heavy rainfall, the brook becomes scoured by flash flooding. Paratypes were collected from similar small streams at high elevations.

Water quality data obtained by using colorimetric analyses of the brook at the type-locality are as follows; pH: 4, hardness: 0, oxygen: 9 ppm. The air temperature was 21°C and the water temperature was 17°C when the analyses were made.

## Supplementary Material

XML Treatment for Hypsilara

XML Treatment for Hypsilara royi

## References

[B1] Brown HP (1981) Key to the world genera of Larinae (Coleoptera, Dryopoidea, Elmidae), with descriptions of new genera from Hispaniola, Colombia, Australia, and New Guinea. The Pan-Pacific Entomologist 57 (1):76-104

[B2] Hinton HE (1940) A monographic revision of the Mexican water beetles of the family Elmidae. Novitates Zoologicae 42(2): 19–396

[B3] Kodada J Jäch MA (1999) *Roraima carinata* gen. *et* sp.nov. and *Neblinagena doylei* sp.nov., two Larainae from Mount Roraima, Venezuela (Coleoptera: Elmidae). Entomol. Probl. 30(1): 13–30

[B4] Kodada J Jäch MA (2005) Elmidae Curtis, 1830. In: Beutel RG Leschen RAB (Eds) Handbook of Zoology. Volume IV. Arthropoda: Insecta. Part 38. Coleoptera, Beetles. Walter de Gruyter, Berlin, Germany, 471–496

[B5] Kukalová-Peck J Lawrence J (1993) Evolution of the hind wing in Coleoptera. The Canadian Entomologist 125:181-258 doi: 10.4039/Ent125181-2

[B6] Sharp D (1882) Biologia Centrali-Americana. Insecta, Coleoptera, Haliplidae, Dytiscidae, Gyrinidae, Hydrophilidae, Heteroceridae, Parnidae, Georissidae, Cyathoceridae I(2): 1–144 Royal Dublin Society, Dublin

[B7] Spangler PJ (1985) A new genus and species of riffle beetle, *Neblinagena prima*, from the Venezuelan tepui, Cerro de la Neblina (Coleoptera, Elmidae, Larinae). Proceedings of the Entomological Society of Washington 87(3): 538–544

